# Supplementary material for: Searching for Topological Semi‐Complete Bandgap in Elastic Truss Lattices
Source: Adv Sci (Weinh). 2025 Sep 12;12(44):e11884. doi: 10.1002/advs.202511884 (PMC12667474; doi:10.1002/advs.202511884)
Supplement: Supplementary file 1 — Supporting Information [file ADVS-12-e11884-s001.docx]

Searching for topological semi-complete bandgap in elastic truss lattices

Supplementary Materials

Yiran Hao^1,*^, Dong Liu^2,5*†^, Liyou Luo^2,*^, Jialu Mu^3,*^, Hanyu Wang^1^, Zibo Liu^4^, Jensen Li^2,†^, Zhihong Zhu^1,†^, Qinghua Guo^3,†^ and Biao Yang^1,†^

^1^College of Advanced Interdisciplinary Studies, National University of Defense Technology, Changsha 410073, China

^2^Department of Physics, Hong Kong University of Science and Technology, Clear Water Bay, Hong Kong, China.

^3^School of Physics and Electronics, Hunan University, Changsha 410082, China.

^4^State Key Laboratory of Tribology in Advanced Equipment (SKLT), Department of Mechanical Engineering, Tsinghua University, 100084, Beijing, China

^5^School of Microelectronics and Physics, Hunan University of Technology and Business, Changsha 410205, China

**,**

**I. The evolution of the elastic truss lattice begins with a diamond lattice structure.**

The design of the elastic meta-crystal starts from a common diamond lattice, as shown in Fig. S1(a), with a six-fold degeneracy at the $\Gamma$ point and an eighth-fold degeneracy at the $R$ point. As three arms of the tetrapod eventually stretch to lie within the same plane (Fig. S1(b) to S1(c)), the six-fold degeneracy at the $\Gamma$ point has been lifted, and a spin-1 Weyl point, marked by a red dot appears. Given that a topological degeneracy invariably exists in pairs with an oppositely charged counterpart, a “charge-2 Dirac point” with fourfold degeneracy emerges at the corner of the BZ (R), indicated by a blue dot. The presence of the spin-1 Weyl point and the charge-2 Dirac point becomes increasingly pronounced within the gap region. In the subsequent step, as shown in Figs. S1(d, e), we refine the band structure by gradually reducing the radius of the pink rods. The optimized design, which achieves the ideal band spectrum, is illustrated in Fig. S1(e).


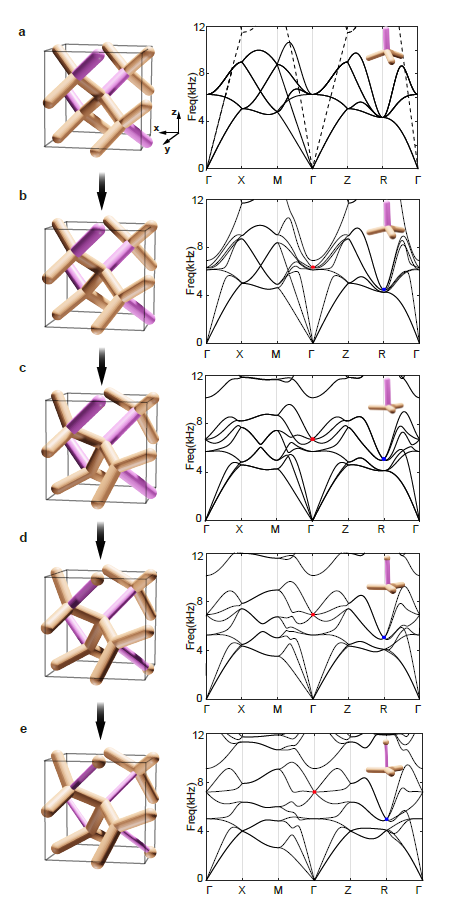


FIG. S1. Schematic view of elastic meta-structures and numerical band spectra. (a)-(e) The evolution progresses from the initial unit cell to the final unit cell that belongs to space group P213 (No. 198).

**II. Eigenmodes of the degenerate flat bands.**

Figure S2 displays the eigenmode displacement fields corresponding to the eightfold degeneracy at the $\Gamma$ point of flat bands in the band structure depicted in Fig. 3(c).


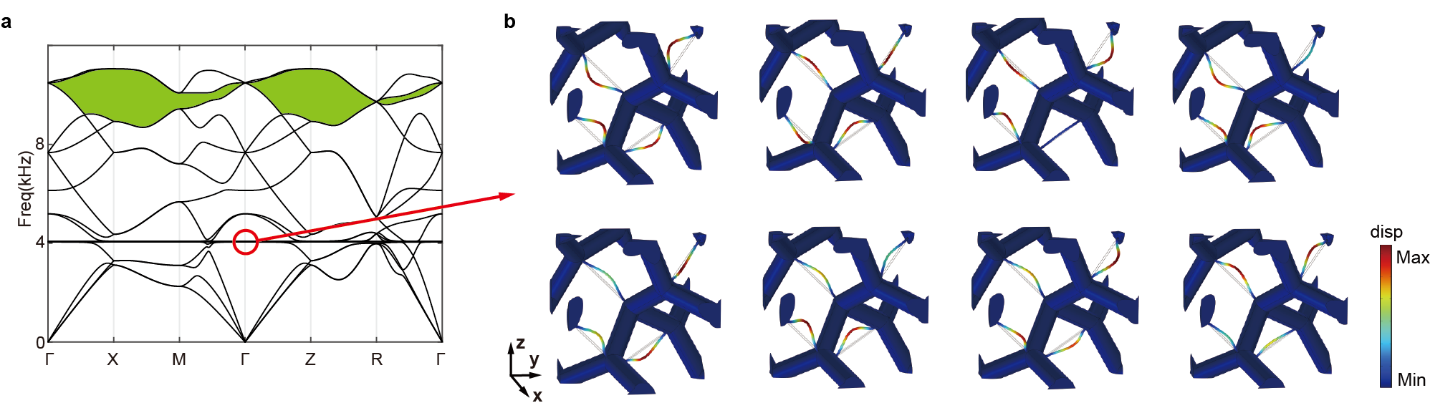


FIG. S2. Eigenmodes of the eight-fold degeneracy at the $\Gamma$ point for flat band. (a) Band structure of the unit cell with $r_{1}=0.25$ mm. (b)Vibration patterns of the displacements on each rod.

**III. Details of the experimental demonstrations.**

Figure S3 illustrates the details of the experiments. To excite the bottom surface states, we affix a piezoelectric transducer near the edge of the bottom surface. Owing to the presence of numerous hollow spaces on the structure's surface, we apply plastic patches to each unit cell to facilitate accurate positioning during detection with a scanning laser vibrometer.


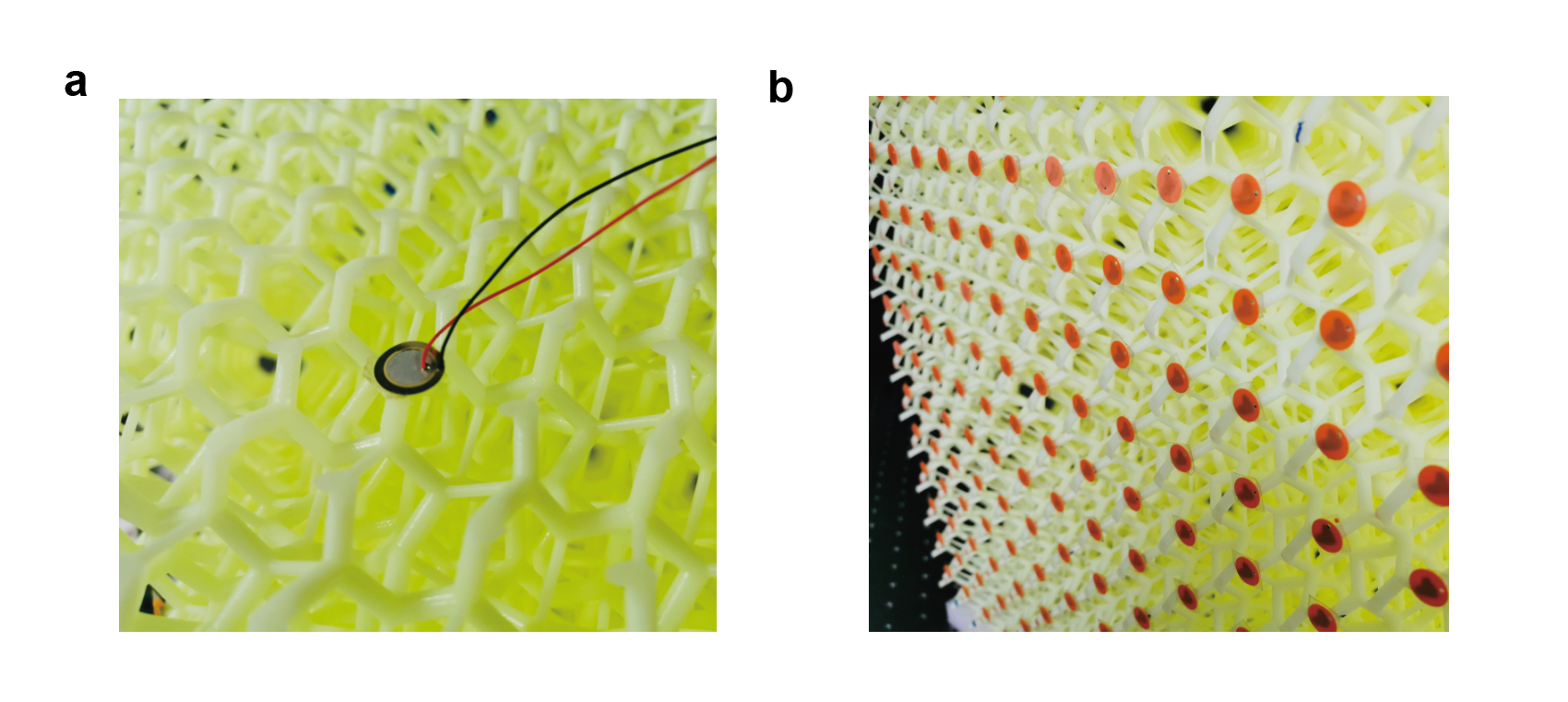


FIG. S3. Experimental sample for the elastic Weyl meta-crystal. (a) The attached piezoelectric transducer near the edge of the bottom surface. (b) Plastic patches affixed to the measured bottom surface.

**IV. Numerical topological surface arcs.**

A striking phenomenon associated with each pair of oppositely charged Weyl points is the emergence of topologically protected surface state arcs, known as Fermi arcs, which connect their projections on the two-dimensional surface Brillouin zone (BZ). Fig. S4 shows how the numerical surface arcs winding around the triple and quadruple degenerate points over frequencies ranging from 6.51 kHz to 7.10 kHz, with the red and blue lines representing the surface states on the top and bottom surfaces, respectively.


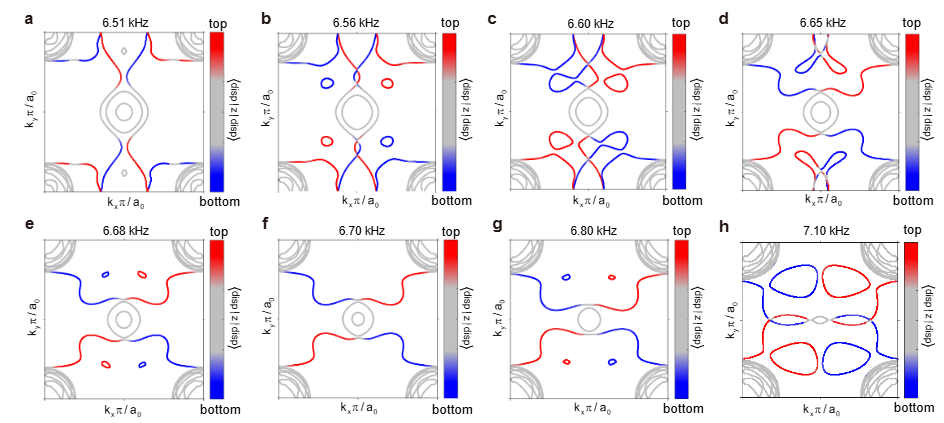


FIG. S4. Numerical Fermi arcs at (a) 6.51 kHz, (b) 6.56 kHz, (c) 6.60 kHz, (d) 6.65 kHz, (e) 6.68 kHz, (f) 6.70 kHz, (g) 6.80 kHz and (h) 7.10 kHz. The color map shows the value of $\left\langle d|z|d \right\rangle$ with $d=\sqrt{u^{2}+v^{2}+w^{2}}$ ($u,v,w$ represent the displacements along the x, y, and z directions).

**V. Experimental demonstrations of elastic topological surface states.**

From the numerical results in Fig. S5(a), our elastic meta-crystal exhibits nontrivial topological gapless surface states within a broad energy gap. Using a scanning laser vibrometer, we experimentally measure the out-of-plane displacement fields on the bottom surface and subsequently perform a 2D Fourier transformation to obtain the projected surface spectra within surface BZ. As shown in Fig. S5(b), surface dispersions within the band gap region at approximately 7 kHz are observed along $\bar{Y}$ to $-\bar{X}$, albeit with a slight deviation in the slope compared with the simulation results, which is attributed to the attached patches and unavoidable fabrication errors. In other frequency regions, additional nontrivial surface dispersions emerge within the gaps, which is consistent with the simulated bottom surface spectra (see the blue lines in Fig. S5(a)). In addition, we also experimentally measure the trivial bulk bands, with the excitation source positioned on the counter face of the test surface, as shown in Fig.S5(c).


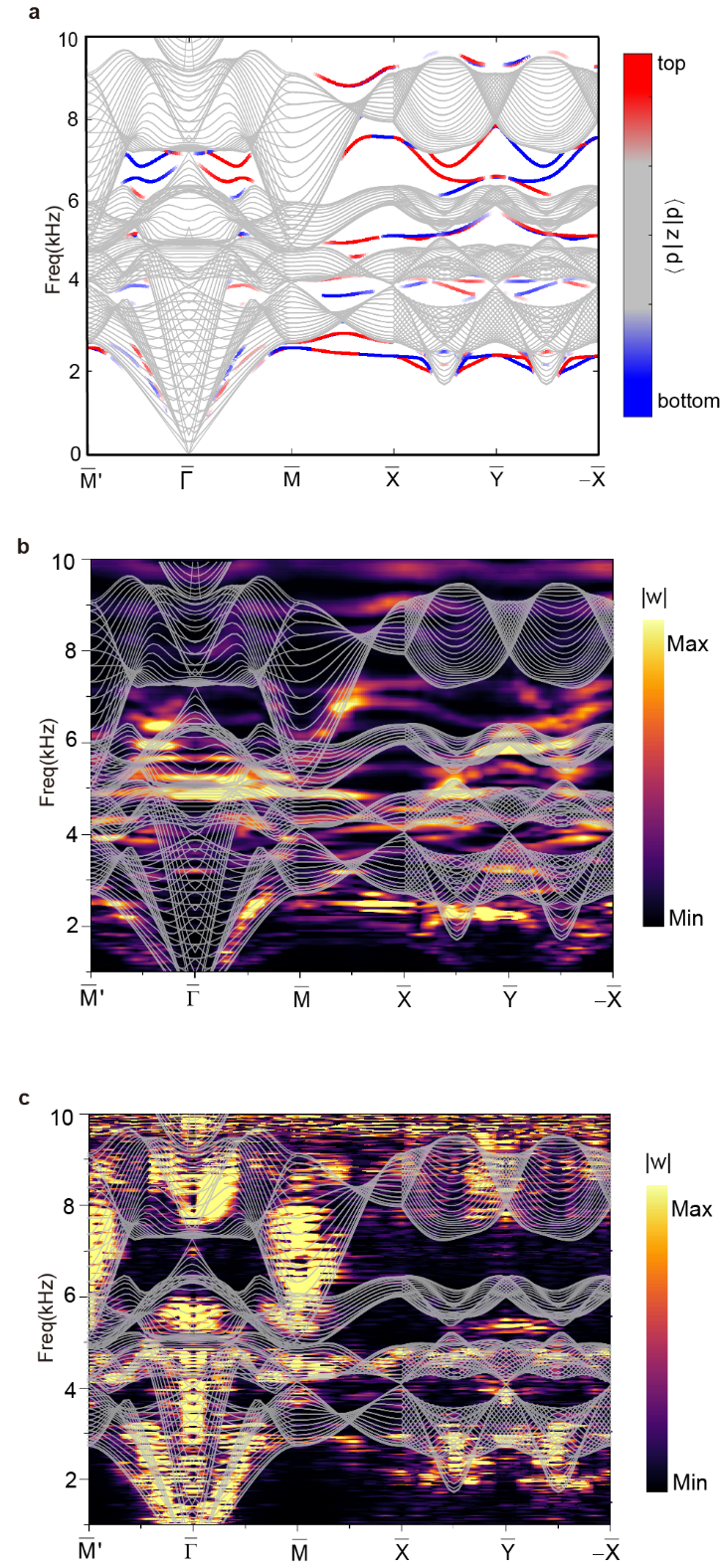


FIG. S5. (a) Surface bands along high-symmetry paths are calculated by setting the free boundary conditions on the top and bottom surfaces (along the z direction). The color map shows the value of $\left\langle d|z|d \right\rangle$ with $d=\sqrt{u^{2}+v^{2}+w^{2}}$ ($u,v,w$ represent the displacements along the x, y, and z directions). (b) Experimental measured topological surface bands. (c) Experimental measured trivial bulk bands.
